# Comparative Clinical-Imaging and Histogenetic Analysis Between Astrocytoma IDH-Mutant Grade 4 and Glioblastoma IDH-Wildtype—Is There Really a Worse One?

**DOI:** 10.3390/diagnostics15040438

**Published:** 2025-02-11

**Authors:** Cristian Ionut Orasanu, Mariana Aschie, Mariana Deacu, Madalina Bosoteanu, Sorin Vamesu, Manuela Enciu, Georgeta Camelia Cozaru, Anca Florentina Mitroi, Sinziana Andra Ghitoi, Ana Maria Cretu, Oana Andreea Ursica, Raluca Ioana Voda

**Affiliations:** 1 Department of Pathology, Clinical Service of Pathology, “Sf. Apostol Andrei” Emergency County Hospital, 900591 Constanta, Romania; 2 Faculty of Medicine, “Ovidius” University of Constanta, 900470 Constanta, Romania; 3 Center for Research and Development of the Morphological and Genetic Studies of Malignant Pathology (CEDMOG), “Ovidius” University, 900591 Constanta, Romania; 4 Department of Anatomy, Academy of Medical Sciences of Romania, 030171 Bucharest, Romania; 5 Department VIII—Medical Sciences, The Romanian Academy of Scientists, 030167 Bucharest, Romania; 6 Department of Genetics, Clinical Service of Pathology, “Sf. Apostol Andrei” Emergency County Hospital, 900591 Constanta, Romania

**Keywords:** astrocytoma, glioblastoma, microvascular density, risk factors, survival

## Abstract

**Background:** Brain tumors pose a significant health threat, leading to high morbidity and mortality rates. Astrocytoma IDH-mutant grade 4 (A4_IDHmt_) and glioblastoma IDH-wildtype (G4I_DHwt_) exhibit similar clinical and imaging characteristics. This study aims to highlight the differences in their clinical evolution and histogenetic aspects with the possible therapeutic impact, as well as the adverse prognostic factors in patient survival. **Methods:** We performed a 10-year retrospective study of grade 4 gliomas, evaluating immunomarkers and FISH tests. We also quantified tumor necrosis and microvascular density. **Results:** A total of 81 cases were identified; 54.32% were A4_IDHmt_. We observed that A4_IDHmt_ patients were younger (34.10% under 50) and had a higher survival rate (4.55%). This group also exhibited a more pronounced microvascular density (*p* = 0.010) and proliferative index (*p* = 0.026). G4_IDHwt_ was associated with larger tumor volumes (94.84 cm^3^ vs. 86.14 cm^3^), lower resectability rates (82.88% vs. 87.67%), and a more significant immature cell population (83.78% vs. 68.18%). In the case of both, the negative risk on survival in the univariate analysis is given by advanced age (A4_IDHmt_: HR = 1.035, G4_IDHwt_: HR = 1.045) and p53 immunopositivity (A4_IDHmt_: HR = 6.962, G4_IDHwt_: HR = 4.680). **Conclusions:** The negative risk factors for A4_IDHmt_ include the rapid onset of clinical symptoms (HR = 2.038), diabetes mellitus (HR = 2.311), arterial hypertension (HR = 2.325), residual tumor (HR = 2.662), increased residual tumor volume (HR = 1.060), increased microvascular density (HR = 1.096), and high tumor necrosis (HR = 1.097). For G4_IDHwt_, the negative risk factors consist of increased residual volume (HR = 1.023), lost PTEN immunoreaction (HR = 33.133), and unmethylated DNA status (HR = 6.765, respectively HR = 20.573). Even if it has more risk factors, A4_IDHmt_ is the lesser evil.

## 1. Introduction

Brain tumors represent a serious threat to health, leading to high rates of morbidity and mortality. Their challenging locations and aggressive growth patterns make them particularly lethal. Gliomas account for approximately 30% of all primary brain tumors and contribute to 80% of malignant brain tumor cases. They account for the majority of deaths caused by primary brain tumors, with an annual rate of 4.42 per 100,000 [1]. Grade 4 gliomas primarily account for these statistics, as indicated by the 5-year relative survival rate of 6.9% [1,2].

Astrocytoma IDH-mutant grade 4 (A4_IDHmt_) is defined as a diffuse infiltrative astrocytic glial proliferation with an IDH1 or IDH2 mutation associated with tumor necrosis, microvascular proliferation, or the homozygous deletion of the CDKN2A/B gene, or any combination of the above characteristics [3]. To these can be added the loss or mutation of the ATRX gene, the TP53 mutation, and alterations in the CDK4, CCND2, MET, MYC, MUCN, PDGFRA, RB1, PIK3R1, and PIC3CA genes [4].

Glioblastoma IDH-wildtype (G4_IDHwt_) is defined as a diffuse infiltrative glial astrocytic proliferation without an IDH gene mutation that associates one or more of the following characteristics: tumor necrosis, microvascular proliferation, TERT promoter mutation, EGFR gene amplification, and/or numerical alteration of chromosomes +7/−10 [5]. Alterations in the TP53, B-RAF V600E, GATA4, FGFR1, MGMT, WT1, and PTEN genes can be added to these [6].

The clinical manifestations of the two tumors are represented by intracranial hypertension (IH), headache, motor deficits, epilepsy, altered general status, confusion, speech deficit, and sensory deficits. Imaging presents as heterogeneous, infiltrative, ring-enhancing intraparenchymal lesions with central necrosis and a peritumoral edema. On a magnetic resonance imaging examination, the center of the lesion is hypointense on T1-weighted images, and the lesion is surrounded by an edema that appears hyperintense on T2-weighted images and with fluid-attenuated inverse recovery (FLAIR). The microscopic appearance is also similar. It consists of a glial proliferation with a marked pleomorphism, increased mitotic activity, microvascular proliferation, and necrosis that can be geographic or accompanied by cellular palisading [7,8,9].

The two tumors present similar clinical, imaging, histopathological, and sometimes molecular aspects, which are mentioned above, as well as high mortality rates. These aspects make us ask the question: Is there really one that is worse? To answer this question, the aim of this study is to identify the small differences, which will prove essential in current clinical practice, through the interaction of pathogenic mechanisms with radiochemotherapeutic ones. We also want to make a comparative analysis of the adverse prognostic factors involved in the evolution of the two tumors in order to correctly evaluate the unfavorable prognosis and the already low survival of the patients.

## 2. Materials and Methods

We conducted a retrospective study for a period of 10 years of patients diagnosed with a central nervous system tumor hospitalized at the Constanta County Emergency Clinical Hospital. The inclusion criteria consisted of a histopathological and immunohistochemical diagnosis of supratentorial grade 4 gliomas (according to the latest WHO criteria from 2021) and patients over 18 years old. The exclusion criteria consisted of recurrences and cases diagnosed at autopsy.

Imaging examinations were performed before neurosurgical intervention and were aimed at localization, size and volume, peritumoral edema, and midline shift. Total or subtotal resection type was evaluated postoperatively. Based on the remaining tumor volume, the resectability rate was calculated.

The immunohistochemical examinations were performed at the Center for Research and Development of the Morphological and Genetic Studies of Malignant Pathology (CEDMOG). Immunohistochemical tests used the markers IDH1 R132H (H09, ready-to-use, HIER-DAB method), Ki-67 (SP6, ready-to-use, HIER-DAB method), p53 (SP5, ready-to-use, HIER-DAB method), PTEN (6H2.1 ready-to-use, HIER-DAB method), Nestin (10C2, ready-to-use, HIER-DAB method), and MGMT (MT 23.2, dilution 1:750, HIER-DAB method). The IDH1 R132H marker was evaluated for case stratification. The Ki-67 reference index was calculated as the percentage of positive nuclei after counting 10 HPF of at least 1000 nuclei. An expression above 10% was considered positive for p53. The PTEN reaction was evaluated at the cytoplasmic and nuclear levels. The Nestin marker was assessed using the glial cells’ cytoplasmic immunoreaction (strong, moderate, or weak) and reactivity (positive or negative) in endothelial cells. Slides were scanned with a TissueScope LE120 Slide Scanner (Huron Digital Pathology, Ontario, CA, USA), and captures were made of 10 microvascular hotspot areas with dimensions of 1 mm^2^ each. The total number of capillary vessels identified by two pathologists was divided by 10, resulting in the average number of vessels per 1 mm^2^. MGMT was assessed at the nuclear level in glial tumor cells and then stratified into <10%, 10–50%, and >50%.

CDKN2A gene alterations were performed using fluorescent in situ hybridization (FISH) at CEDMOG. The cytogenetic evaluation used ZytoLight SPEC CDKN2A/CEN 9 Dual Color Probe probes (Bremerhaven, Bremen, Germany). Fluorescent signals of the preparations were calculated in 100 tumor nuclei using a fluorescence microscope, Zeiss Axio Imager 2 (Zeiss Gmbh, Jena, Germany). In cells without abnormalities, two green (CDKN2A gene region) and two orange signals (CEN 9 probe) were observed. In the cells with deletions, fewer orange signals were seen, and in the case of amplifications, more orange signals were seen.

A statistical data analysis was performed in SPSS Statistics Version 26 (IBM Corporation, Armonk, NY, USA). Indicators of central tendency and variability were used. A univariate data analysis was performed using the Chi-square test, Fisher’s exact test for categorical data, the Mann–Whitney U Test, and the Kruskal–Wallis H Test for continuous variables. To establish the association of the data, we used the Pearson Correlation Coefficient. The receiver operating characteristic (ROC) and area under the curve (AUC) values were used to establish the accuracy of the parameters. The sensitivity and specificity of the parameters are the optimal cut-off point as the value that maximizes the area under the ROC curve. Survival estimates were determined over a 5-year follow-up period and were calculated using the Kaplan–Meier method. The survival differences between groups were analyzed by applying the log-rank test. Hazard ratios (HRs) were appreciated using a Cox regression analysis. Results were considered statistically significant at a *p*-value of <0.05.

All patients signed the informed consent form at the time of hospitalization, and the ethics opinion was obtained from the local ethics commission (Ethics Commission of the Constanta County Emergency Hospital, Constanta, Romania).

## 3. Results

### 3.1. Clinical Features and Survival Outcomes

**Figure 1:**
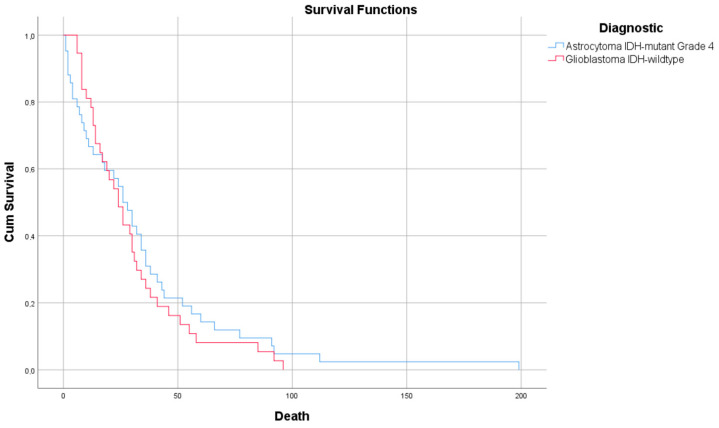
Kaplan–Meier survival graphic showing increased survival for A4_IDHmt_ compared with G4_IDHwt_ (*p* = 0.527).

We identified 81 cases of grade 4 supratentorial gliomas, 54.32% of which were A4_IDHmt_. The most frequent complaints in both cases were motor deficits, headaches, and cognitive disorders. Regarding the patients’ history, no associations of comorbidities with the presence of pathology were observed (Table 1). In the case of astrocytomas, the median survival was 34.45 (1–199) weeks, and in the case of glioblastomas, 29.68 (6–96) weeks (*p* = 0.527) (Figure 1).

**Figure 2:**
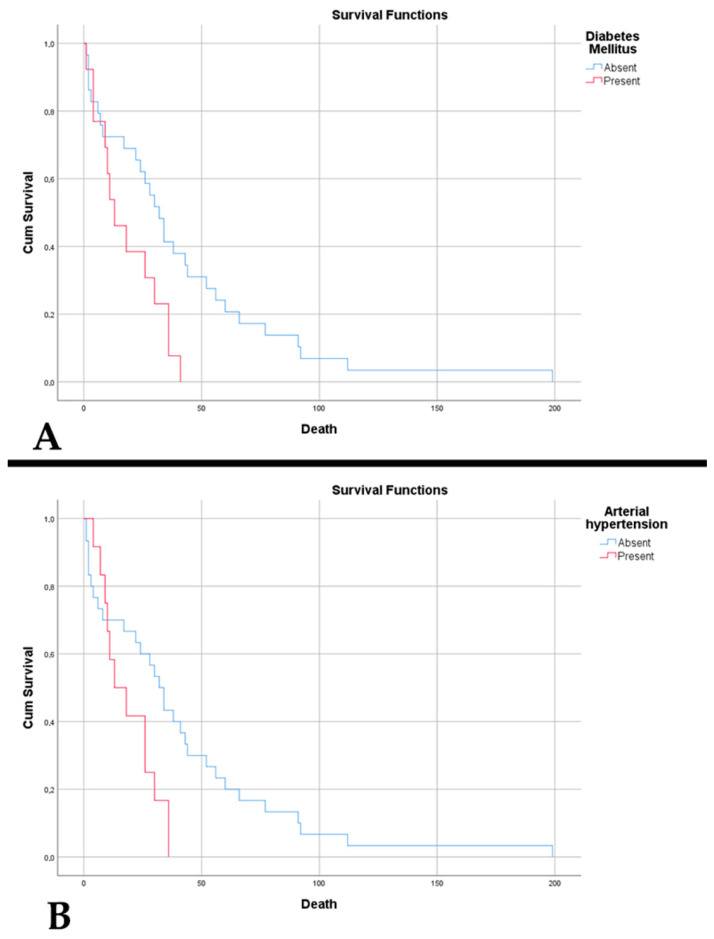
(**A**) Kaplan–Meier survival graph showing decreased survival in patients with A4_IDHmt_ who had diabetes mellitus (*p* = 0.019). (**B**) Kaplan–Meier survival graph showing decreased survival in patients with A4_IDHmt_ who had arterial hypertension (*p* = 0.023).

In A4_IDHmt_, we observed an association of low age with intracranial hypertension (*p* = 0.037). We noted that ages younger than 52 years are susceptible to developing intracranial hypertension, with a sensitivity of 88.9% and a specificity of 66.7% (*p* = 0.037, AUC = 0.727). Those whose symptoms had an onset under 2 weeks had a lower survival (24.20 weeks vs. 48 weeks, *p* = 0.032). In the case of female patients, a statistically significant correlation was observed with the presence of motor deficits or paresis (*p* = 0.025). The presence of motor deficits was associated with the presence of cognitive disorders (*p* = 0.005). Regarding comorbidities, we observed that patients who presented with arterial hypertension (AH) and diabetes mellitus (DM) had a lower survival: 18.83 weeks vs. 40.70 weeks (*p* = 0.023), and 18.39 weeks vs. 41.66 weeks (*p* = 0.019), respectively (Figure 2).

In G4_IDHwt_, we observed that patients over 50 had a lower median survival (24.94 weeks vs. 60 weeks, *p* = 0.013). Similar to astrocytomas, patients with AH and DM had a lower survival but without statistical significance (*p* = 0.517 and *p* = 0.138, respectively).

The negative prognostic factors identified for astrocytomas are advanced age, acute onset of clinical manifestations, and comorbidities (hypertension and diabetes mellitus). Regarding glioblastomas, the only negative risk factor is advanced age (Table 2).

### 3.2. Imaging Characteristics and Tumor Metrics

Most gliomas (53.09%) were located in the left cerebral hemisphere. The most frequently affected lobes were temporal (23.46%), frontal (13.58%), and parietal (13.58%). Table 3 shows the distribution of the imaging data according to tumor entity.

In A4_IDHmt_, we observed a difference in the occurrence of lesions according to sex and hemisphere. Thus, we observed a predominance of right-hemisphere involvement in men and left-hemisphere involvement in women (*p* = 0.033). The presence of the lesion in the right hemisphere was associated with the development of epileptic seizures (*p* = 0.021), and left hemisphere involvement was related to the presence of cognitive disorders (*p* = 0.034). An increased tumor diameter and an increased volume were associated with an increased residual volume (*p* < 0.001, *p* < 0.001, respectively). In contrast, only an increased tumor volume was associated with a reduced time to death (*p* = 0.019). The maximum diameter and tumor volume do not show any statistical prediction for the occurrence of clinical manifestations (*p* > 0.050), resulting in the fact that the tumor’s location and not its dimensions/volume determine the occurrence of manifestations. A tumor diameter >3.1 cm has a sensitivity of 90% and a specificity of 75% for the occurrence of peritumoral edema (*p* = 0.007, AUC = 0.913). The presence of tumor residue and a low resectability rate were associated with a short overall survival (*p* = 0.018, *p* < 0.001, respectively). Patients who underwent complete trimodal treatment had a much higher survival (49.18 weeks vs. 5 weeks, *p* < 0.001). We observed the same aspect in the absence of tumor residue (66.33 weeks vs. 25.76 weeks, *p* = 0.017).

In G4_IDHwt_, we observed associations between tumor location and clinical manifestations. The presence of epileptic seizures and IH were associated with right-hemisphere localization (*p* = 0.042, *p* = 0.012, respectively), and cognitive impairment was associated with left-hemisphere tumor localization (*p* = 0.001). We observed that a midline shift >7.5 mm has a sensitivity of 75% and a specificity of 60% for developing IH (*p* = 0.029, AUC = 0.725). A more remarkable midline shift correlated with a presentation to the doctor after 2 weeks from the onset of symptoms (*p* = 0.019). Patients under 50 had a higher resectability rate (*p* = 0.008). Both increased maximum tumor diameter and increased volume were associated with increased residual volume (*p* = 0.003, *p* < 0.001, respectively). Patients who received complete trimodal treatment had a significantly superior survival (34.80 weeks vs. 7.71 weeks, *p* < 0.001).

The univariate and multivariate analyses of the data identified both risk factors and protective factors for both tumor entities (Table 4).

### 3.3. Histogenetic and Molecular Findings

Both tumors were characterized by a high percentage of tumor necrosis, an increased microvascular density (MVD), and a high proliferative index. In most cases, the population that dominated the morphological picture is immature according to the immunoreaction to Nestin (Table 5).

**Figure 3:**
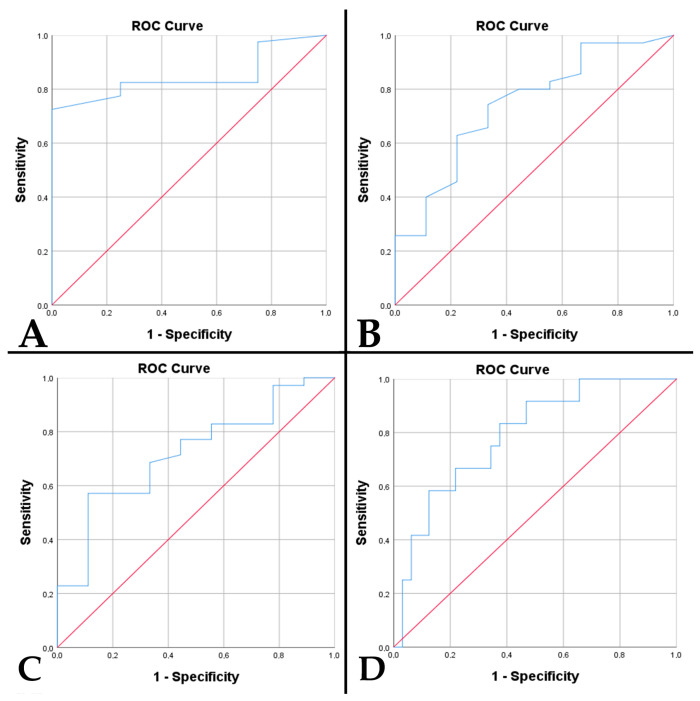
The ROC curve underlines predictable values (Red line represents the ROC curve for a random guess. The blue lines represent the distribution of the cases.) in cases of A4_IDHmt_ of: (**A**) percentage of tumor necrosis with the presence of peritumoral edema (*p* = 0.023, AUC = 0.847); (**B**) percentage of tumor necrosis with p53 immunopositivity (*p* = 0.027, AUC = 0.741); (**C**) microvascular density with p53 immunopositivity (*p* = 0.045, AUC = 0.719); (**D**) microvascular density with absent PTEN immunoreaction (*p* = 0.003, AUC = 0.789).

In A4_IDHmt_, a high percentage of tumor necrosis was associated with the presence of peritumoral edema (*p* = 0.019). However, no associations were observed with an increased tumor diameter (*p* = 0.071) or midline shift (*p* = 0.284). A percentage of >19.5% tumor necrosis has a sensitivity of 82.5% and a specificity of 75% in predicting the occurrence of tumor edema (*p* = 0.023, AUC = 0.847) (Figure 3A). Also, in the case of patients with diabetes mellitus, this was associated with a higher percentage of tumor necrosis (*p* = 0.017). The high percentage of tumor necrosis was associated with incomplete trimodal treatment (*p* < 0.001), an increased residual volume (*p* < 0.001), a low resectability rate (*p* < 0.001), and implicitly with a shorter time to death (*p* < 0.001).

Tumor necrosis is directly associated with MVD (*p* < 0.001). Also, an increased necrotic percentage is correlated with p53 positivity (*p* = 0.025). A cut-off of >24.5% necrosis is predictive for p53 immunopositivity, with a sensitivity of 74.3% and a specificity of 66.7% (*p* = 0.027, AUC = 0.741) (Figure 3B). The absence of a PTEN immunoreaction correlated with an increased necrotic percentage (*p* < 0.001). Thus, a necrotic percentage of over 35.5% is predictive of the absence of a PTEN immunoreaction, with a sensitivity of 83.3% and a specificity of 68.7% (*p* < 0.001, AUC = 0.874). We observed differences in the relationship between DNA methylation status, quantified using MGMT, and the necrotic percentage (*p* = 0.022). The highest percentage of tumor necrosis was identified in the case of MGMT > 50%, followed by immunoreactivity of 10–50% and <10%. In cases with MGMT > 50%, a poor prediction of the cut-off of > 38% necrosis was identified (sensitivity 62.5%, specificity 71.4%, *p* = 0.036, AUC = 0.692). Like MGMT, the highest percentage of tumor necrosis was observed in CDKN2A gene amplifications, followed by gene deletions and normal status (*p* = 0.008). A percentage of over 44.5% is predictive of gene amplification with a sensitivity of 100% and a specificity of 81.5% (*p* = 0.034, AUC = 0.870).

In female patients with A4_IDHmt_, a higher MVD was observed (*p* = 0.043). An increased MVD was associated with a lower resectability rate (*p* < 0.001), thus with an increased residual volume (*p* < 0.001), incomplete trimodal treatment (*p* < 0.001), and a shorter time to death (*p* < 0.001). MVD is increased in the presence of a positive p53 (*p* = 0.043) and the absence of PTEN immunoreactivity (*p* = 0.003). Thus, an MVD of > 46.75 vessels/mm^2^ has an acceptable prediction for p53 immunopositivity, with a sensitivity of 68.6% and a specificity of 66.7% (*p* = 0.045, AUC = 0.719) (Figure 3C). An MVD of > 52.25 vessels/mm^2^ has an acceptable prediction for PTEN absence with a sensitivity of 75% and a specificity of 65.6% (*p* = 0.003, AUC = 0.789) (Figure 3D). We observed differences in the relationship between the DNA methylation status and MVD (*p* = 0.022). An increased MVD is identified in the case of an MGMT of >50%, followed by 10–50% immunoreactivity and <10%. Similarly, in CDKN2A gene amplifications, the richest tumor vascularization is observed, followed by gene deletions and normal status (*p* = 0.008). An MVD of > 65.85 vessels/mm^2^ shows a sensitivity of 100% and a specificity of 87.8% for gene amplification (*p* = 0.009, AUC = 0.959).

In A4_IDHmt_, we observed a statistically significant difference between cellular maturation measured using nestin immunointensity and the proliferative index (*p* = 0.015). This consists of a high index in cells with reduced immunointensity, followed by moderate and high immunointensity. A similar aspect was observed in the case of MGMT, where the highest proliferation values were recorded in the >50% category, followed by 10–50% and then <10% (*p* < 0.001). Thus, a Ki-67 > 55% is predictive of an MGMT of > 50% with a sensitivity of 100% and a specificity of 92.9% (*p* > 0.001, AUC = 0.980). A proliferative index of >77.5% has an excellent predictability for death (sensitivity 100%, specificity 83.3%, *p* = 0.049, AUC = 0.917).

**Figure 4:**
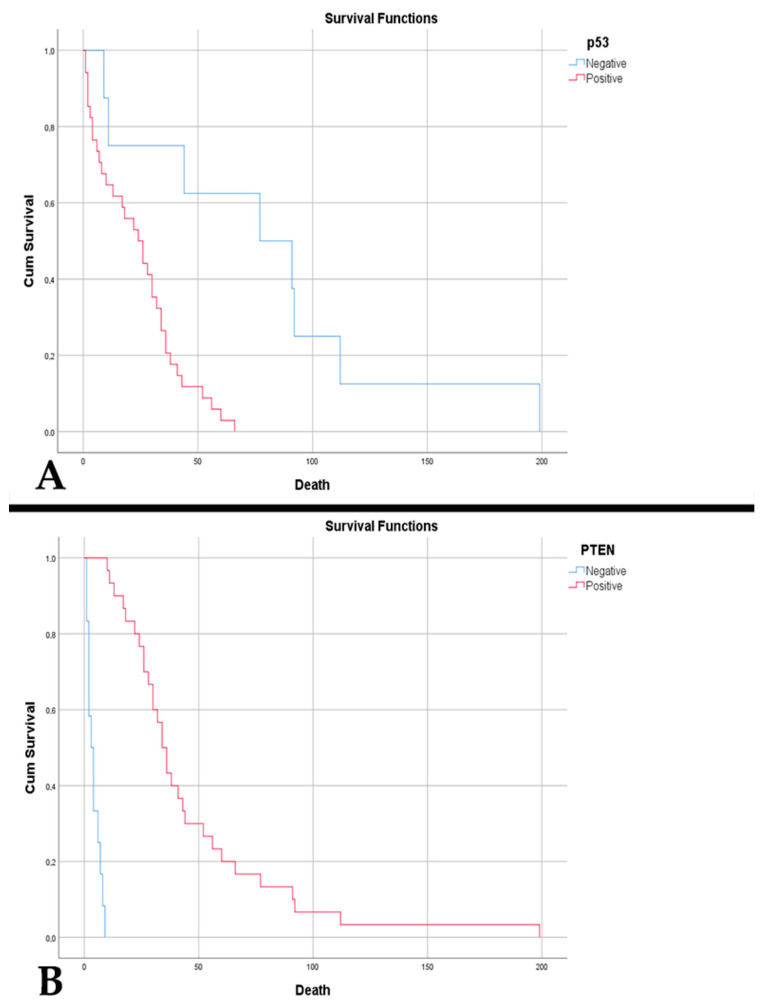
(**A**) Kaplan–Meier survival graph showing decreased survival in patients with p53 immunopositivity in cases of A4_IDHmt_ (*p* < 0.001). (**B**) Kaplan–Meier survival graph showing decreased survival in patients with absence of PTEN immunoreaction in cases of A4_IDHmt_ (*p* < 0.001).

P53-positive cases were correlated with an increased residual volume (*p* = 0.048). P53 immunopositivity was reflected in lower patient survival—23.88 weeks vs. 79.38 weeks (*p* < 0.001) (Figure 4A). The same aspect was noted in an absent PTEN immunoreaction, 4.08 weeks vs. 46.60 weeks (*p* < 0.001) (Figure 4B). Also, a negative PTEN was correlated with an increased residual volume (*p* = 0.016).

We observed statistically significant differences between cellular immaturity and MGMT, consisting of the correlation of low immunointensities and an absent Nestin immunoreaction with MGMT > 50% (*p* = 0.002). Statistically significant differences were observed between the DNA methylation status and CDKN2A gene status. Thus, an immunoreaction of >50% was correlated with gene deletion and 10–50% with gene amplification (*p* = 0.016). We also observed a difference between the gene status and the tumor resection percentage, the lowest percentage being in gene amplification, followed by deletion and normal status (*p* = 0.045). Differences were observed between patient survival and CDKN2A gene status: for amplifications, 9.67 weeks; for deletions, 22.70 weeks; and for normal status, 50.74 weeks (*p* = 0.002).

In G4_IDHwt_, we observed statistically significant associations of tumor necrosis with microvascular density (*p* < 0.001) and with proliferative index (*p* < 0.001). An increased percentage of tumor necrosis was associated with the mature cell population (*p* = 0.038). We identified differences between the necrotic percentage and CDKN2A gene status (*p* < 0.001). The highest percentage of tumor necrosis was noted in gene amplifications, followed by gene deletions and normal gene status. A similar aspect was observed in the case of MVD (*p* < 0.001). Increased MVD was associated with an increased proliferative index (*p* = 0.006) and an increased residual volume (*p* = 0.030). We found that MVD also reflects its effects in the patient’s clinic. Thus, an MVD of >26.55 vessels/mm^2^ has a sensitivity of 89.5% and a specificity of 61.1% for predicting the delayed onset of symptoms (*p* = 0.014, AUC = 0.737).

**Figure 5:**
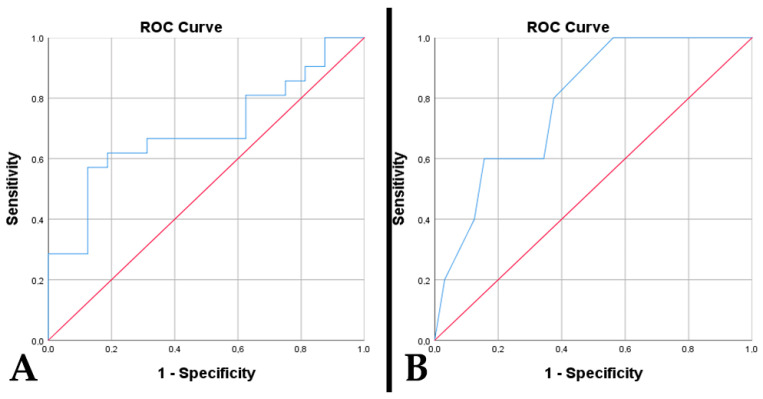
The ROC curve underlines predictable values (Red line represents the ROC curve for a random guess. The blue lines represent the distribution of the cases.) in cases of G4_IDHmt_ of: (**A**) microvascular density for p53 immunopositivity (*p* = 0.046, AUC = 0.693); (**B**) cell proliferation index with absent PTEN immunoreaction (*p* = 0.041, AUC = 0.788).

**Figure 6:**
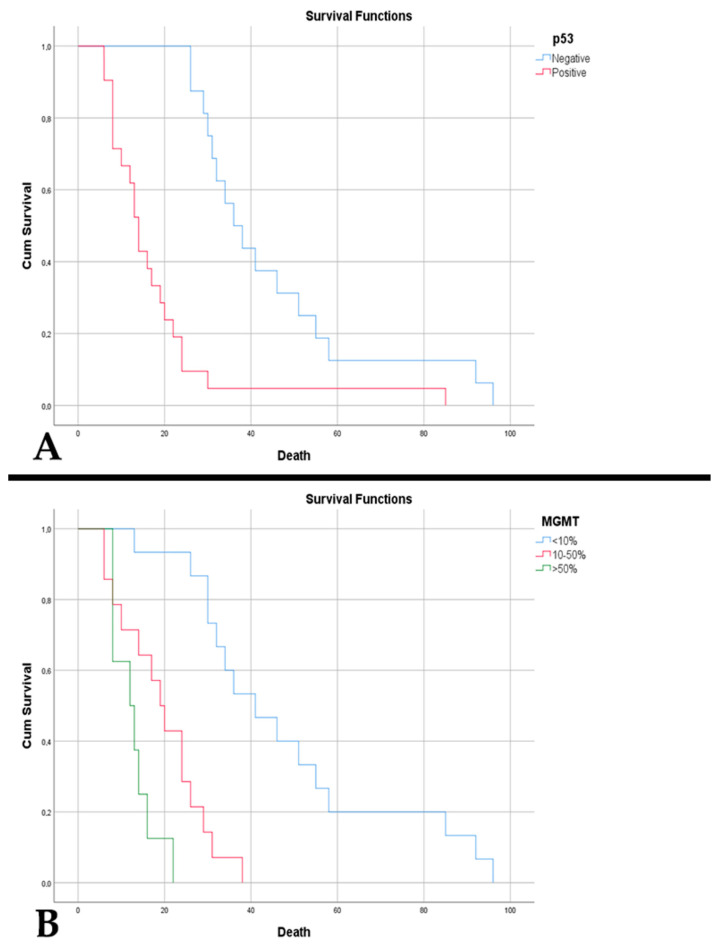
(**A**) Kaplan–Meier survival graph showing decreased survival in patients with p53 immunopositivity in cases of G4_IDHwt_ (*p* < 0.001). (**B**) Kaplan–Meier survival graph showing survival in patients with the three levels of immunoexpression of DNA methylation status in cases of G4_IDHwt_ (*p* < 0.001).

Increased MVD in glioblastomas was associated with p53 immunopositivity (*p* = 0.047). An MVD of >32.25 vessels/mm^2^ was a poor predictor of p53 positivity with a sensitivity of 66.7% and a specificity of 68.7% (*p* = 0.046, AUC = 0.693) (Figure 5A). P53 positivity was associated with older age (*p* = 0.032), incomplete trimodal treatment (*p* = 0.012), and a shorter time to death (*p* < 0.001). This was correlated with MGMT >10% (10–50% and >50%) (*p* < 0.001), as well as with altered CDKN2A gene status (*p* = 0.018). Overall survival was low in p53-positive cases (17.95 weeks vs. 45.06 weeks, *p* < 0.001) (Figure 6A) and those with absent PTEN (7.2 weeks vs. 33.19 weeks, *p* < 0.001).

An absent PTEN response was associated with incomplete trimodal treatment and a shorter time to death (*p* < 0.001, *p* < 0.001, respectively). Negative PTEN and altered CDKN2A gene status were associated with an increased proliferative index (*p* = 0.041, *p* = 0.048, respectively). A proliferative index of >32.5% predicted the absence of PTEN immunoreactivity with a sensitivity of 80% and a specificity of 62.5% (*p* = 0.041, AUC = 0.788) (Figure 5B).

An increased tumor volume in G4_IDHwt_ was associated with the presence of immature cell populations (Nestin negative or weakly positive) (*p* = 0.018). The same aspect of the Nestin immunomarker was also observed in relation to the residual volume (*p* = 0.001). The most abundant MVD identified was in the mature cell population, followed by populations with a high degree of immaturity (*p* = 0.040). We observed a lower survival (28.96 weeks) in cases with abundant immature populations (Nestin intensely positive), in contrast to cases with mature populations (Nestin negative)—36 weeks (*p* = 0.011).

DNA methylation status in glioblastomas, with MGMT values above 10% (10–50% and >50%), correlated with an age of patients over 50 years (*p* = 0.016), incomplete treatment (*p* = 0.030), and a lower resectability rate (*p* = 0.034). A residual volume of over 19.72 cm^3^ was predictive of MGMT of > 50% with a sensitivity of 75% and a specificity of 79.3% (*p* = 0.029, AUC = 0.754). In cases with MGMT of > 50%, the median survival was 12.63 weeks compared with MGMT expression of 10–50%: 19.43 weeks and MGMT of < 10%: 48.33 weeks (*p* < 0.001) (Figure 6B).

In G4_IDHwt_, CDKN2A gene status exerted a negative effect on tumor necrosis and microvascular density. Thus, a necrotic percentage of >34.5% has a sensitivity of 90% and a specificity of 70.4% for the prediction of gene deletion (*p* = 0.005, AUC = 0.807), and a percentage of >48.5% has a sensitivity of 100% and a specificity of 79.4% for the prediction of gene amplification (*p* = 0.023, AUC = 0.902). Also, an MVD of >36.15 vessels/mm^2^ has a sensitivity of 100% and a specificity of 77.8% for gene deletion (*p* < 0.001, AUC = 0.926), and an MVD of > 59.05 vessels/mm^2^ has a sensitivity of 100% and a specificity of 82.4% for gene amplification (*p* = 0.040, AUC = 0.863).

The presence of tumor necrosis and microvascular density play a critical role in the unfavorable prognosis of patients with astrocytoma, to which is added the status of the CDKN2A gene. In the case of glioblastomas, the negative impact is demonstrated through PTEN expression (PTEN retained: HR = 0.030, PTEN lost: HR = 33.133) and DNA methylation status (Table 6).

## 4. Discussion

### 4.1. The Integration of Clinical Aspects

According to the latest WHO classification of central nervous system tumors (2021), grade 4 gliomas are represented by A4IDHmut and G4_IDHwt_ [10]. The global incidence of these tumors is varied, with differences depending on their geographical distribution or histology. A higher incidence is found in Europe and North America [2,11]. Thus, A4_IDHmt_ has an age-adjusted annual incidence of 0.07/100,000, and G4_IDHwt_ has a higher age-adjusted annual incidence of 1.74/100,000. The availability of neurosurgical intervention can essentially justify these variations, the quality of the neuropathological data provided, or the complete recording of data in cancer registries [1,2].

These tumors can occur at any age, but A4_IDHmt_ occurs most frequently in the age range of 31–51 years, while G4_IDHwt_ occurs more frequently in the age range of 57–72 years [12]. In the present study, patients with A4_IDHmt_ had an older age at presentation, but the age difference between the two entities was not significant (57.86 years vs. 60.24 years). It is noteworthy that we identified more patients under 50 years of age in A4_IDHmt_, in accordance with the literature. Also, a predilection for the male sex was observed in both entities, A4_IDHmt_ 1.3:1, G4_IDHwt_ 1.6:1, respectively [10,13]. The same trend was observed in our study.

Clinical manifestations have an onset that can be rapid and can be confused with a stroke or can evolve over several months [14]. This study’s particularity is represented by quantifying the onset period of symptoms. From this, we observed a faster onset of A4_IDHmt_. This aspect can be explained by the correlation between the low age of the patients and the development of intracranial hypertension, which is supported by the ROC curve. On the other hand, the later onset of G4_IDHwt_ can be explained by the association with the displacement of the midline and the increase in microvascular density. Both situations mentioned above need time to develop, especially in the conditions in which we observed that MVD is much lower than A4_IDHmt_.

Signs and symptoms can be caused by three mechanisms. The first is represented by the direct effect of tissue destruction through tumor necrosis. In this case, the most frequent manifestations consist of focal neural deficits (paresis), cognitive disorders, and psychiatric syndromes (especially in frontal locations) [14,15]. This aspect was noted in A4_IDHmt_ through the association of cognitive disorders with motor deficits.

The second mechanism consists of increased intracranial pressure, either by increasing tumor mass or peritumoral edema [14,15,16]. This increase in intracranial pressure was associated with young age in the present study. We found no explanation for this aspect in the literature except in cases of idiopathic intracranial hypertension [17]. The evocative symptom of IH is headache with progressive severity and without a specific pattern [14,15,16]. Something that was particularity identified in the multivariate analysis of A4_IDHmt_ cases was identifying headaches as a protective factor. The specialized literature did not focus on evaluating headaches as a risk factor of gliomas. This was limited to correlations that were either therapeutic or in relation to the clinical phenotype. Only the study by Russo M et al. identified headache as a protective factor in the case of tumor localization in the left hemisphere [18,19]. Another frequently encountered sign that most often resolves once the peritumoral edema subsides is the presence of cognitive disorders [20].

The third mechanism depends on the tumor location and is usually manifested through the presence of epileptic seizures [14,15,16]. We noted two similar aspects represented by cognitive dysfunctions in tumors in the left hemisphere and the presence of epileptic seizures in tumor localization in the right hemisphere. Also, in the multivariate analysis of G4_IDHwt_ cases, we observed the protective role of preoperative epilepsy. The studies of Ge H et al. and Li L et al. noted the same protective role of the epilepsy factor reflected in increased survival, respectively increased survival, and progression-free survival [21,22]. In our study, patients with G4_IDHwt_ who presented with epileptic seizures had a longer survival (37.38 weeks vs. 27.56 weeks) but without a statistically significant correlation (*p* = 0.377). It should also be mentioned that the first study aggregates all grade 4 gliomas, and the second considers high-grade diffuse gliomas.

Among comorbidities, we observed the negative influence of AH and DM as prognostic factors on the survival of patients with A4_IDHmt_. The mechanisms of action of hypertension in these tumors are still unknown. The literature is scarce in terms of analyzing hypertension as a comorbidity in brain tumors. The effect of antihypertensive medications on the central nervous system is suspected. Loop diuretics and thiazides contain amides and amines that are carcinogenic to the nervous system [23]. In contrast, patients undergoing antiangiogenic treatment, with bevacizumab, who developed hypertension secondary to treatment, showed a higher survival and progression-free survival [24,25]. The study by Aboubechara JP et al. showed that hypertension was associated with poor survival in the case of G4IDHwt. They concluded that the presence of hypertension along with other factors related to the metabolic syndrome (insulin resistance, hyperglycemia, obesity, and dyslipidemia) affects the prognosis of patients with glioblastoma through a chronic inflammatory status. This microclimate in which proinflammatory cytokines are found, as well as the activation of the NF-κB and STAT3 pathways, potentiates cell proliferation, invasiveness, and resistance to apoptosis. Thus, tumor progression is accelerated and the effects of classical treatments are diminished [26]. To these aspects, a specific postoperative complication for patients with hypertension can be added: the occurrence of spontaneous intracranial hemorrhage leading to the death of patients [27].

The relationship between DM and grade 4 gliomas is controversial. Some studies have not observed a relationship between the two, and others have noted a low overall survival of patients. Also, this survival seems to be negatively influenced by the gross total resection and adjuvant treatment [28]. Some studies have observed some thresholds for glycemic values. These were either >112 mg/dL and >180 mg/dL or >174 mg/dL, to be associated with decreased survival [29,30]. The interaction between high-grade gliomas and diabetes mellitus is mediated by a series of membrane transport proteins: glucose transporters (GLUTs). GLUT3 supports aerobic glycolysis through the astrocytic uptake of glucose and its transport across the blood–brain barrier. In high-grade gliomas, GLUT3 expression is upregulated to support the nutritional support of proliferation. In contrast, patients with diabetes mellitus and glioblastoma show a low expression, further forcing the tumor microenvironment towards anaerobic metabolism. Thus, this metabolic adaptation leads to a stem-like cancer cell phenotype with a greatly increased aggressiveness and proliferative rate [31]. Therefore, the control of hyperglycemia and diabetes mellitus are essential for the survival of patients with gliomas. Metformin, a biguanide used in the treatment of type 2 diabetes mellitus, has major clinical importance. Its effects consist of lowering blood glucose, inhibiting mTOR signals, triggering apoptosis and autophagy through p53 and p21, destroying cancer stem cells, and preventing angiogenesis. However, in glioblastomas, monotherapy did not improve progression-free survival or overall survival. Instead, together with temozolomide, it acts synergistically, leading to the eradication of tumor cell chemoresistance, the potentiation of apoptotic activity, and the inhibition of proliferation [32,33].

In the case of this non-specific symptomatology, the first clue to the diagnosis is given by the imaging examination. Magnetic resonance imaging is the preferred modality for the presumptive diagnosis and characterization of these tumors. This technique has the advantage of providing high-resolution structural details, precise tumor localization, and dimensions. Other methods used are computed tomography and positron emission tomography. The first has the advantage of availability, a lower cost, and fast scanning times. Still, it has two major disadvantages: patient exposure to radiation and lower accuracy than MRI on soft tissues. Positron emission tomography is a useful tool in the diagnosis, prognosis, and monitoring of gliomas, as well as in providing data on their biological aspects [34,35,36].

### 4.2. The Integration of Immaging Aspects

Most frequently, these tumors are located in the frontal lobe, followed by temporal and parietal [37]. A similar aspect is present in this study, with the same three preferential locations but in a different order. We observed that the only correlation of tumor topography is with symptomatology. From the results of this study, we can state that the tumor’s location and not its dimensions determines the appearance of clinical manifestations.

Over time, various methods of measuring the tumor using an imaging examination have been tried: axial diameters, area, and volume using 3D ellipsoid methods or 3D volume using semi-automatic segmentation methods. The highest accuracy is shown using the technique of calculating the volume using semi-automatic 3D segmentation methods, but it does not show correlations with other data about the tumor [38,39,40]. However, the tumor volume is of particular importance in the application of radiotherapy. With the help of imaging techniques, a delineation based on the resected cavity together with any tumor residue without including peritumoral edema can be obtained. In addition to the optimal and targeted administration of radiotherapy, another role of delineation is to protect the organs at risk: the optic chiasm, the optic nerve, normal brain tissue, and the brainstem [41]. Other studies consider the volume measurement technique more faithful to low-grade gliomas. In the case of grade 4 gliomas, there are discrepancies in patient survival caused by the intratumoral necrotic volume and the extension of the lesions [38,39,40].

In G4_IDHwt_, we observed an increase in tumor volume in association with the presence of immature cell populations. An explanation for this phenomenon is based on the Warburg effect that occurs in the hypoxic processes present in these tumors. This effect, together with the wildtype status of isocitrate dehydrogenase, will upregulate the expression of NHE1. It is responsible for extracellular matrix remodeling, angiogenesis, and, implicitly, the decrease in overall survival. Thus, NHE1 inhibitors have the role of decreasing tumor volume and invasiveness, and increasing overall survival [42,43].

In the case of A4_IDHmt_, the tumor diameter is associated with peritumoral edema, even indicating a threshold value of >3.1 cm. The development of a peritumoral edema is of particular importance, as it is responsible for the development of intracranial hypertension, neurological symptoms, and cerebral herniation [44], aspects that were partially found in the present study. In the present study, the appearance of IH is also correlated with the displacement of the midline. Thus, a displacement of the midline by >10 mm is responsible for the appearance of IH. The study by Wach J et al. considers the same cut-off of the midline displacement in predicting a low survival and a bad postoperative evolution. However, this aspect does not consider the IDH status [45]. However, a low edema index has a positive effect on disease control but not on patient survival. Studies have shown that the volume of peritumoral edema correlates more with the aggressiveness of gliomas than tumor size [44,46]. Our study strengthens the data from the literature through a multivariate analysis, where we found that in A4_IDHmt_ and G4_IDHwt_, increased volume, and, respectively, increased tumor diameter, are positive factors in patient survival. This aspect is also supported by the study by Raj R et al., who noted an increase in the survival of patients with increased tumor volume. This fact was observed in academic hospitals and was due to a higher resectability rate in large tumors compared with small ones [47].

We observed that the higher resectability rate correlated with age under 50 and a lower rate for people over 50. This aspect can be attributed to cerebral senescence. Senescence supports cancer progression through the coordinated action of the p53 and p16 tumor suppression pathways. Initially, p53 stabilization occurs with the transcription of the cyclin-dependent kinase inhibitor; later, control is taken over by p16, leading to an increased accumulation of senescent cells that evade immune system clearance [48]. This would explain the association in G4_IDHwt_ of p53 immunopositivity with advanced age and a shorter period until death. Similar to the study by Meel M et al., the association of IDH1 status with p53 was translated into a lower survival of patients, especially in the case of IDH1 wildtype and p53 [49]. The advantage of gliomas with a mutant IDH1 status lies in the possibility of using IDH inhibitors. Ivosidenib and vorasidenib prevent the formation of the oncometabolite and intercept its downstream oncogenic activity. Both drugs increase progression-free survival, except in cases of disease enhancement [50].

There is no consensus on the extent of resection to stratify prognostic groups. However, gross total resection is the path to increased overall survival and progression-free survival, compared with biopsies, and partial or subtotal resections [51,52]. Chaichana et al. showed that a resectability rate of 70% with a residual tumor volume of <5 cm^3^ can prolong survival by 3.9 months [53]. In the present study, we observed that for A4_IDHmt_ and G4_IDHwt_, an increased tumor volume is a significant risk factor for mortality. In addition, in cases of A4_IDHmt_, the resection rate and the type of excision complete the prognostic picture.

After neurosurgical resection, treatment is completed by radiotherapy and chemotherapy [54]. These must be in accordance with the guidelines of the European Association of Neuro-Oncology, which also considers the tumors’ molecular profile [55]. Radiotherapy begins 3–5 weeks after resection, and 50–60 Gy is administered in 1.8–2 Gy per day, with a 15-mm margin around the total gross volume. Hypofractionated radiotherapy is used in patients >65 years and those with a poor prognosis [41,56]. Chemotherapy is based on alkylating agents such as temozolomide, especially for IDH-mutant gliomas. Alkylating agents from the nitrosoureas class, such as carmustine, lomustine, nimustine, and fotemustine, can also be used. These have a lower penetration and more frequent and severe side effects. In recurrent glioblastomas, bevacizumab, an anti-VEGF antibody, can also be used, but it does not have significant benefits for overall survival [56]. Some of our patients received the classic radiotherapy treatment regimen and temozolomide. Those who managed to complete the trimodal treatment had a significantly superior survival.

### 4.3. The Involvement of Hypoxic Effects in Morphology

Necrosis, vascular proliferation, and cellular immaturity are part of the effects of hypoxia in these tumors. Hypoxia is caused by an insufficient nutrient supply and consists of a decrease in the partial pressure of O_2_ to 5–9 mmHg (normal brain tissue 25–40 mmHg) and a pH lower than 6.8 (normal brain tissue 7.1). Hypoxia also promotes a metabolism based on anaerobic glycolysis, maintaining an acidic pH. Under the influence of hypoxia, hypoxia-inducible factor-1 (HIF-1) activates proangiogenic genes. The neovascularization process is a multistep one that begins with gathering tumor cells around pre-existing vessels. They proliferate after vascular regression occurs with the formation of necrosis and the formation of a new vascular network [57,58,59]. Thus, the newly formed vessels do not provide adequate oxygen pressure, maintaining and developing the necrosis phenomena. Under these conditions, cancer cells adapt their metabolism to survive. Moreover, perinecrotic cells have an increased tendency to proliferate [57,60]. Exposure to a hypoxic environment upregulates canonical stem cell genes, such as CD133 and Nestin, facilitating plasticity towards a stem-like state. Also, the acidic environment promotes the glioma stem cell phenotype. It induces changes in cell metabolism through changes between glycolysis and oxidative respiration (increased glycolysis and the inhibition of oxygen consumption) [59]. These cells, whether true stem cells or progenitor cells with a stemness status, will be found in perinecrotic or perivascular niches [60]. This codependent relationship between necrosis, microvascular proliferation, cell proliferation, and cell maturity was also identified in the present study through the identified correlations.

In G4_IDHwt_, separating the effects of necrosis and hypoxia is impossible, and the classic microscopic features—microvascular proliferation and perinecrotic palisading—are closely linked to both. Thus, necrosis represents an overshoot of the blood supply and is associated with rapid growth and poor prognosis. Therefore, the need is increased in conditions of rapid and uncontrolled cell division leading to diffusion-limited hypoxia [61]. The present study supports the association of necrosis with microvascular density and the proliferative index.

Studies have shown the association of hypoxia with the loss of PTEN expression as bring involved in thrombosis and necrosis in high-grade gliomas [62]. Hayati N et al. found associations between the PTEN mutation, the absence of an immunoreaction, advanced age, and tumor necrosis [63]. Our study only observed the association between necrosis and the absence of PTEN reaction in cases of A4_IDHmt_. Moreover, we identified a cut-off of necrosis that predicts the PTEN immunoreaction. The importance of the PTEN gene status is also reflected in therapeutic management. Thus, patients with PTEN deficiency may have a chance by using poly adenosine diphosphate-ribose polymerase (PARP) inhibitors. Veliparib (PARP1 inhibitor) associated with temozolomide represents an effective method in glioblastomas. In addition, the combination of pamiparib (PARP inhibitor) and temozolomide associated with radiotherapy and platinum derivatives is being studied in high-grade IDH-mutant astrocytomas [64]. In the analysis of the spatio-temporal dynamics of hypoxia in gliomas performed by Grimes DR et al., they observed a cellular concentration with the overexpression of p53 around necrotic areas [65]. We identified the association in cases of A4_IDHmt_, in which we observed a prediction of the necrotic percentage for p53 positivity.

An aspect identified by us, which derives from hypoxic phenomena, is represented by the correlation of tumor necrosis with peritumoral edema. The overexpression of the HIF family in the perinecrotic area leads to VEGF stimulation with angiogenesis and the production of immature and dysfunctional vessels. This aspect determines the rupture of the blood–brain barrier and the formation of a marked peritumoral edema [33,66]. Regarding this correlation, we identified a minimum threshold for predicting the appearance of a peritumoral edema concerning tumor necrosis. The association of the destruction of the blood–brain barrier with the properties of the stem-like population (self-renewal and a multilineage differentiation capacity) leads to increased therapeutic resistance. In addition, mouse models have shown that stem-like cells resist much better to cell death caused by radiotherapy and chemotherapy [59].

Recent studies have observed that HIF-1 inhibition can reduce MGMT levels in glioblastoma stem cells [67]. Thus, a negative or low expression is associated with increased patient survival, while moderate and high reactions are associated with chemoresistance and reduced survival [68]. Methylation status is particularly important in resistance to temozolomide (TMZ). Under TMZ treatment, MGMT can remove the methyl group from O6-methylguanine, thereby neutralizing drug-induced DNA damage and reducing the overall efficacy of TMZ. Therefore, the epigenetic status of MGMT has been established as a surrogate marker of intrinsic TMZ resistance [69]. It should be noted that negative or low expression represents a methylated gene status, while the other reactions represent an unmethylated status [70]. In our A4_IDHmt_ cases, the relationship with the effects of hypoxia is very close. We observed statistically significant correlations with tumor necrosis, microvascular density, proliferative index, and cellular immaturity, as well as threshold values of tumor necrosis, microvascular density, and the proliferative index in relation to gene expression status. These associations may represent major premises regarding the treatment with terameprocol. In addition to antiviral effects, it also possesses antiangiogenic, antineoplastic effects and induces reversible G2/M cell cycle arrest in mammalian cell lines without significant cytotoxicity [71].

The results of Shamsara J et al. highlighted an aspect also noted in the present study regarding the DNA methylation status and p53 overexpression. In G4_IDHwt_, MGMT methylation is frequently encountered and is associated with mutant p53 expression [72]. This unfortunate association is one of the causes of chemoresistance to alkylating agents, through the inability to induce apoptosis [73]. Normally, p53 upregulation downregulates MGMT expression and vice versa in these tumors. Based on the analysis of previous studies, Shamsara J et al. noted the mechanism of association between gene methylation and the p53 gene status. Thus, in the early stages of glioblastoma development, p53 is stabilized using posttranslational modifications. Subsequently, it is upregulated causing MGMT downregulation. With pathogenic progression, this downregulation gradually induces p53 mutation in cells, and low levels of wildtype p53 will continue to lead to deregulation of the methylation pattern. Even if wildtype p53 levels recover, they can only intervene in MGMT downregulation because the methylation process is irreversible [72]. This association, as well as the involvement of the p53 gene in cellular senescence, explains the identification of the correlation between age over 50 years and the immunoexpression of >10% of the DNA methylation status. Notably, the effects of DNA methylation are also reflected in medical practice from the pretherapeutic stage. The study by Alafandi A et al. observed an association between pre-radiotherapy tumor volume and the methylated status of the gene [74]. In the present study, we only observed the relationship with the resectability rate, not with the pretumor volume in G4_IDHwt_. In addition, we identified a threshold value of the residual tumor volume in the case of immunoexpression of > 50%, which is reflected in the low survival rate of these patients.

The mutational status of the TP53 gene is associated with tumor progression in glioblastomas. The most common deregulation in the p53 pathway is represented by the deletion of the CDKN2A/ARF locus [75]. In the present study, we also observed an association of p53 expression with the alteration of the CDKN2A gene. The role of the CDKN2A gene deletion in both diagnosis and prognosis is well known, especially in A4_IDHmt_ [76]. However, its connection with tumor necrosis has not been studied much. In the present study, we noted that regardless of the status of the IDH gene, associations with gene abnormalities are observed. In addition, in A4_IDHmt_, a percentage of over 44.5% tumor necrosis is predictable for gene amplification, and in G4_IDHwt_, values of > 34.5% and > 48.5% have predictability for deletion and gene amplification, respectively.

Around the areas of necrosis, the cell population has a characteristic palisade arrangement. Glial stem cells represent the majority of the population in these areas. An essential characteristic of these is that they not only survive the hypoxic environment in that area, but the HIF family induces their presence. Thus, the perinecrotic niche develops [77]. Nestin is a marker that manages to highlight these glial precursor cells. Its expression highlights the dedifferentiated status, invasive potential, high degree of malignancy, and increased cell motility [78,79]. As noted by An S et al. and in our study, the importance of this marker is greater in high-grade IDH wildtype gliomas [80]. In cases of G4_IDHwt_, we observed lower survival rates in cases of increased Nestin immunointensities. Also, the effect of immature populations was reflected in an increased tumor volume, as well as in an increased residual volume. The role of stem cells can explain this aspect. Namely, they represent the essential support in the pathogenesis of treatment resistance and relapses [77]. In cases of A4_IDHmt_, we observed an association between the proliferative rate and the immature cell population. Thus, an average rate of 36.74% was associated with immature cells, with this value gradually increasing to 58.57% in mature, Nestin-negative cells (*p* = 0.015). This aspect has also been noted in the literature, but in cases of a mouse model as recurrence after temozolomide therapy [77]. Also, the expression of stem-like cells is important in radiotherapy resistance. In addition to Nestin, these cells express the transcription factor forkhead box M1 (FoxM1) and overexpress SOX2. In xenograft models of glioblastoma, the FoxM1-Sox2 pathway has been shown to be involved in the radiotherapy response [81].

The lack of cellular oxygen leads to the stimulation and release of proangiogenic factors. Similarly, the hypoxic environment causes cancer stem cells to differentiate into endothelial progenitor cells. Thus, abnormal and functionally immature vessels are born, characterized by variable lumen diameters, hyperpermeability, and irregular branching [82]. Imaging studies have identified a more active angiogenesis process in the case of the wildtype status of the IDH1 gene, which translates into a more prosperous vascular network. They found that the lower vascularity in A4_IDHmt_ explains the increased survival in these cases [83]. The present study observed a much richer vascularization in A4_IDHmt_ cases. The microvascular density in these cases correlated with the resectability rate, residual volume, and patient deaths. Even though G4_IDHwt_ MVD is lower, its effects are reflected in the onset of symptoms, even with the predictive value, on cell proliferation and residual volume. These correlations make the increase in MVD a risk factor in the death of patients with grade 4 gliomas, a result that adheres to the analysis performed by Fan C et al. [84].

As in our study, the study by Sipos TC et al. identified a higher vascular proliferation in A4_IDHmt_. They identified a higher MVD in p53 wildtype cases (<10% positivity) [85]. Comparatively, we observed the opposite. Moreover, we identified predictive values of MVD on p53 status in both tumor entities.

An interesting aspect was noted by Hiller-Vallina S et al., which consists of the association of estrogen receptor 1 (ESR1) expression and angiogenesis. ESR1 was upregulated in highly vascular tumor areas. This can be explained by the ESR1-associated pathways involved in pericyte migration, cell adhesion, and VEGF signaling. Thus, necroinflammatory changes predominated in the group of men with low ESR1 expression; in the groups of women and men with increased ESR1 levels, vessels without fragility that would predispose to necroinflammatory phenomena were observed [86]. This aspect of hormonal involvement in the maintenance of the angiogenic process is supported by the present study through the association identified between female sex and increased MVD.

The PTEN gene can suppress angiogenesis by inhibiting the nuclear translocation of MDM2. Thus, the PI3K-Akt-PTEN pathway exerts its function as a regulator of angiogenesis but also in the stromal response to angiogenic stimuli. Another critical role of PTEN in glioblastomas is to reduce hypoxia by inhibiting HIF-1 [87]. The present study strengthens the relationship of PTEN with hypoxic consequences in grade 4 gliomas. This aspect is reflected by the increase in microvascular density to >52.25 vessels/mm^2^ in A4_IDHmt_ and cell proliferation to >32.5% in G4_IDHwt_ in cases with an absent PTEN response.

The role of the PTEN gene is controversial in the prognosis of grade 4 gliomas. However, its mutations seem to be an important factor in the pathogenesis of gliomas [88]. It is postulated that alterations of the CDKN2A gene precede mutations of the PTEN gene [89]. PTEN mutations represent an event in the malignant process and may affect the therapeutic efficacy in these tumors, especially in cases of wildtype IDH [90]. In the present study, we noted a decrease in survival in both types of tumors in the absence of a PTEN immunoreaction. This aspect can also be highlighted by the associations with residual volume in A4_IDHmt_ and with CDKN2A gene abnormalities for G4_IDHwt_. In addition, in G4_IDHwt_, the absence of the reaction represents a negative risk factor for patient survival in the univariate and multivariate analyses.

A particular aspect that we identified consists of the differences between microvascular density and alterations in the CDKN2A gene. By quantifying the number of vessels, the status of the gene can be inferred with different sensitivities and specificities in both types of gliomas. This aspect, associated with the relationship that we identified between necrosis and the CDKN2A gene, helps to confirm the perspectives of the study by Appay R et al. in supporting the correlation between CDKN2A gene deletion and MVD and/or necrosis as negative risk factors for overall survival and progression-free survival in the case of mutant IDH gliomas without 1p/19q codeletion [91]. CDKN2A gene alterations, especially deletions, not only have the role of classifying astrocytic gliomas in histopathological grade 4 but also have their effects on patient survival. In clinical practice, cases of A4_IDHmt_ have been observed, with a much worse prognosis than G4_IDHwt_. Thus, it was identified that gene deletions are much more involved in overall survival and progression-free survival than the IDH gene status [92,93]. This aspect explains the low survival in our group of patients with A4_IDHmt_ who had abnormalities in the CDKN2A gene and the identification of abnormalities as negative prognostic factors in their survival. It has also been shown that the inhibition of CDKN2A leads to increased cell viability and decreased sensitivity to carmustine [94]. This alkylating agent has been shown to be effective in increasing progression-free survival and overall survival in glioblastomas, especially in combination with temozolomide [95]. Thus, according to the aforementioned data, we can say that restoring CDKN2A expression may provide an effective solution in the development of targeted therapies.

By corroborating the previous data on the effects of hypoxia with DNA methylation status and the PTEN and CDKN2A genes, the associations identified with the Ki-67 proliferative index are explained. These aspects are of greater importance in cases of A4_IDHmt_, where we identified threshold values of Ki-67 concerning patient survival.

The limitations of this study are represented by its retrospective nature, the need for a larger group of patients, and the fact that the analysis of the DNA methylation status could not be performed using DNA extraction or PCR amplification. In contrast, the strengths of this study consist in highlighting some similarities between the two different pathological processes and some differences that indicate an unfavorable prognosis. Also, identifying the threshold values of necrosis, microvascular density, and the proliferative index were decisive in highlighting the mode of action of the hypoxic microclimate on the status of various genes involved in patient survival.

## 5. Conclusions

Until recently, the two tumor types were considered identical, presenting clinical, imaging, histopathological, and even some molecular similarities. The significant differences that we identified consisted in the appearance of A4_IDHmt_ at a younger age (under 50 years), a higher microvascular density, and a more pronounced cellular proliferation.

In the case of both entities, the negative risk on survival is related to advanced age, increased microvascular density, p53 immunopositivity, and the alteration of the CDKN2A gene. Only for A4_IDHmt_, the negative risk factors are the appearance of clinical manifestations within 2 weeks, diabetes mellitus, hypertension, the presence of tumor residue, increased residual volume, reduced resectability rate, and the presence of a high percentage of tumor necrosis. In comparison, in G4_IDHwt_, the negative risk factors consisted of the presence of an increased residual volume, the absence of a PTEN immunoreaction, and an immunohistochemical expression indicating an unmethylated status of the DNA (immunoreaction of >10%). Even if we identified several risk factors for A4IDHmt, due to the association of patient survival with the currently existing therapeutic possibilities, we can state that astrocytomas are the lesser evil.

## Figures and Tables

**Table 1:** The clinical–evolutionary aspects of the group of patients.

Clinical Aspects	Astrocytoma IDH-Mutant (*n* = 44)	Glioblastoma IDH-Wildtype (*n* = 37)	*p*-Value
Age:			
• Average (min–max)	57.86 (37–82)	60.24 (20–81)	0.232
• Patients <50 years (%)	34.10	13.51	0.040
Gender (%):			0.965
• Female	45.45	45.95	
• Male	54.55	54.05	
The onset of symptoms (%):			0.821
• <2 weeks	51.22	48.65	
• >2 weeks	48.78	51.35	
Clinical manifestations (%):			
• Motor impairments/paresis	65.91	59.46	0.646
• Headache	47.73	35.14	0.269
• Cognitive impairments	43.18	37.84	0.656
• Intracranial hypertension	20.45	32.43	0.309
• Psychiatric syndromes	20.45	13.51	0.558
• Balance and coordination disorders	15.91	21.62	0.574
• Epileptic seizures	13.64	21.62	0.388
Comorbidities (%):			
• Diabetes mellitus	29.55	27.03	0.802
• Arterial hypertension	27.27	40.54	0.242
• Other tumors	9.09	8.11	0.875
• Survival rate (%)	4.55%	0%	0.498

**Table 2:** Risk factors derived from clinical aspects are identified for each tumor entity.

	Astrocytoma IDH-Mutant						Glioblastoma IDH-Wildtype					
	Univariate Analysis			Multivariate Analysis			Univariate Analysis			Multivariate Analysis		
	HR	CI 95%	*p-*Value	HR	CI 95%	*p-*Value	HR	CI 95%	*p-*Value	HR	CI 95%	*p-*Value
Age	1.035	1.005–1.067	0.022	1.010	0.962–1.059	0.691	1.045	1.007–1.084	0.019	1.061	1.001–1.125	0.048
Gender	1.220	0.652–2.282	0.534	1.763	0.738–4.207	0.202	1.312	0.644–2.672	0.455	1.750	0.727–4.212	0.212
The onset of symptoms (<2 weeks)	2.038	1.044–3.978	0.037	5.214	1.751–15.524	0.003	1.110	0.574–2.145	0.757	1.313	0.525–3.282	0.560
Motor impairments/paresis	0.668	0.346–1.290	0.230	0.404	0.109–1.499	0.176	1.263	0.633–2.521	0.508	0.949	0.350–2.574	0.918
Headache	0.630	0.329–1.208	0.165	0.331	0.136–0.805	0.015	1.101	0.553–2.191	0.785	0.707	0.237–2.105	0.533
Cognitive impairments	0.667	0.350–1.271	0.218	0.562	0.172–1.839	0.341	0.844	0.426–1.673	0.627	0.447	0.147–1.358	0.156
Intracranial hypertension	2.073	0.946–4.542	0.069	1.122	0.329–3.824	0.854	0.898	0.447–1.803	0.763	0.534	0.181–1.577	0.256
Psychiatric syndromes	0.794	0.375–1.681	0.546	0.598	0.129–2.776	0.512	0.551	0.191–1.593	0.271	0.456	0.118–1.770	0.257
Balance and coordination disorders	2.069	0.890–4.807	0.091	3.586	0.824–15.606	0.089	1.555	0.700–3.453	0.278	1.964	0.559–6.899	0.292
Epileptic seizures	0.601	0.249–1.448	0.256	0.504	0.119–2.127	0.351	0.704	0.318–1.555	0.385	0.297	0.089–0.995	0.049
Diabetes mellitus	2.311	1.116–4.782	0.024	2.505	0.537–11.690	0.243	1.735	0.821–3.669	0.149	1.457	0.428–4.957	0.547
Arterial hypertension	2.325	1.090–4.956	0.029	0.775	0.171–3.510	0.740	1.246	0.634–2.451	0.523	1.364	0.414–4.495	0.610
Other tumors	1.285	0.451–3.663	0.639	2.797	0.531–14.731	0.225	0.708	0.214–2.343	0.572	0.347	0.062–1.940	0.228

**Table 3:** Distribution of imaging data for each tumor entity.

Imaging Aspects	Astrocytoma IDH-Mutant (*n* = 44)	Glioblastoma IDH-Wildtype (*n* = 37)	*p-*Value
Hemisphere (%):			0.656
• Left	50	56.76	
• Right	50	43.24	
Lobe (%):			0.195
• Frontal	18.18%	8.11%	
• Temporal	25%	21.62%	
• Parietal	9.09%	18.92%	
• Occipital	2.27%	0%	
• Fronto-temporal	4.55%	0%	
• Fronto-parietal	18.18%	5.41%	
• Fronto-insular	2.27%	8.11%	
• Temporo-parietal	9.09%	13.51%	
• Temporo-occipital	0%	5.41%	
• Temporo-insular	2.27%	8.11%	
• Parieto-occipital	9.09%	10.81%	
The mean of the maximum diameter (min–max)	50.39 (10–80)	52.92 (20–87)	0.612
Average volume (cm^3^, min–max)	86.14 (0.90–388.94)	94.84 (1.22–324.72)	0.595
Peritumoral edema (%)	90.91	97.30	0.369
Midline displacement (mm)	8.91	8.97	0.783
Residual tumor present (%)	77.27	75.68	0.866
Average resectability rate (%)	87.67	82.88	0.380
Residual tumor volume (cm^3^)	11.02	15.13	0.513
Complete treatment (%)	68.18	81.08	0.213

**Table 4:** Risk factors derived from imaging aspects were identified for each tumor entity.

	Astrocytoma IDH-Mutant						Glioblastoma IDH-Wildtype					
	Univariate Analysis			Multivariate Analysis			Univariate Analysis			Multivariate Analysis		
	HR	CI 95%	*p-*Value	HR	CI 95%	*p-*Value	HR	CI 95%	*p-*Value	HR	CI 95%	*p-*Value
Hemisphere	1.581	0.838–2.984	0.158	1.786	0.820–3.893	0.144	1.173	0.603–2.283	0.637	1.434	0.605–3.396	0.413
Lobes	1.051	0.950–1.163	0.331	0.937	0.822–1.067	0.325	1.009	0.846–1.162	0.903	1.014	0.848–1.214	0.876
Maximum diameter	0.997	0.979–1.015	0.765	1.018	0.965–1.075	0.509	0.984	0.962–1.006	0.153	0.948	0.902–0.996	0.034
Volume	0.997	0.994–1.001	0.179	0.980	0.966–0.995	0.008	1.000	0.995–1.006	0.865	1.007	0.996–1.019	0.231
Peritumoral edema	1.473	0.450–4.825	0.522	0.633	0.136–2.946	0.560	2.805	0.362–21.750	0.324	8.017	0.476–134.968	0.148
Midline displacement	0.998	0.948–1.051	0.952	0.939	0.861–1.024	0.156	0.979	0.922–1.040	0.492	1.043	0.966–1.126	0.282
Residual tumor present	2.662	1.150–6.160	0.022	10.225	2.076–50.367	0.004	1.376	0.640–2.958	0.413	1.289	0.404–4.109	0.668
Resectability rate	0.866	0.817–0.917	<0.001	0.881	0.792–0.981	0.021	0.982	0.962–1.003	0.094	0.995	0.948–1.043	0.824
Residual tumor volume	1.060	1.034–1.087	<0.001	1.186	1.097–1.281	<0.001	1.023	1.004–1.042	0.018	1.023	0.980–1.068	0.305
Complete treatment	0.001	0.000–1.634	0.066	0.000	0–3.747 × 10^68^	0.876	0	0–14,472,349.37	0.480	0	0–3.235 × 10^79^	0.887

**Table 5:** Distribution of histogenetic data for each tumor entity.

Histogenetic Aspects	Astrocytoma IDH-Mutant (*n* = 44)	Glioblastoma IDH-Wildtype (*n* = 37)	*p-*Value
Tumor necrosis (%, min–max)	34.23 (10–69)	32.49 (7–72)	0.605
Microvascular density (vessels/mm^2^, min–max)	49.95 (22.6–70.8)	40.91 (20.6–78.6)	0.010
Ki-67 (%, min-max)	48.75 (15–90)	36.70 (4–85)	0.026
p53 positive (%)	79.55	56.76	0.032
Absent PTEN (%)	27.27	13.51	0.174
Nestin (%):			0.054
• Negative	15.91	2.70	
• Weakly positive	15.91	13.51	
• Moderately positive	15.91	13.51	
• Intensely positive	52.27	70.27	
MGMT (%):			0.350
• >50%	36.36	21.62	
• 10–50%	34.09	37.84	
• <10%	29.55	40.54	
CDKN2A (%):			0.221
• Normal	47.73	64.86	
• Deletion	45.45	13.70	
• Amplification	6.82	2.74	

**Table 6:** Risk factors derived from the histogenetic aspects identified for each tumor entity.

	Astrocytoma IDH-Mutant						Glioblastoma IDH-Wildtype					
	Univariate Analysis			Multivariate Analysis			Univariate Analysis			Multivariate Analysis		
	HR	CI95%	*p-*Value	HR	CI95%	*p-*Value	HR	CI95%	*p-*Value	HR	CI95%	*p-*Value
Tumor necrosis	1.097	1.063–1.132	<0.001	1.058	0.998–1.122	0.060	1.007	0.989–1.025	0.435	1.035	0.984–1.088	0.181
Microvascular density	1.096	1.057–1.137	<0.001	1.092	1.022–1.167	0.009	1.009	0.993–1.026	0.264	0.934	0.872–0.999	0.046
Ki-67	0.999	0.978–1.011	0.859	0.961	0.925–0.997	0.038	1.003	0.989–1.017	0.679	0.989	0.960–1.019	0.463
p53 positive	6.962	2.035–23.820	0.002	13.244	2.042–85.880	0.007	4.680	2.239–9.781	<0.001	20.459	2.823–148.271	0.003
PTEN retained	0	0–19.374	0.154	0	0–1.116 × 10^85^	0.889	0.030	0.004–0.213	<0.001	0.022	0.002–0.287	0.004
Nestin												
• Weakly positive	1.362	0.415–4.475	0.610	3.935	0.862–17.969	0.077	0.720	0.096–5.389	0.749	8.333	0.320–217.183	0.202
• Moderately positive	1.724	0.535–5.550	0.361	2.087	0.596–7.309	0.250	3.953	1.350–11.574	0.012	15.140	1.910–120.041	0.010
• Intensely positive	1.204	0.446–3.255	0.714	2.460	0.665–9.096	0.177	0.449	0.151–1.335	0.150	0.309	0.053–1.787	0.189
MGMT												
• 10–50%	0.599	0.267–1.339	0.212	2.993	0.852–10.511	0.087	6.765	2.579–17.741	<0.001	8.672	2.055–36.600	0.003
• >50%	1.410	0.629–3.158	0.404	1.187	0.146–9.642	0.873	20.573	5.745–73.672	<0.001	28.678	3.591–229.058	0.002
CDKN2A												
• Deletion	2.612	1.278–5.339	0.009	1.648	0.525–5.177	0.392	1.833	0.860–3.910	0.117	2.842	0.274–29.420	0.381
• Amplification	6.793	1.757–26.271	0.005	1.533	0.194–12.125	0.686	2.482	0.726–8.486	0.147	67.028	2.841–1581.559	0.009

## Data Availability

Data are contained within the article.
